# Recurrent Cardiac Myxoma Treated by Orthotopic Heart Transplantation: A Case Report and Literature Review of Heart Transplantation for Primary Cardiac Tumor

**DOI:** 10.1155/2018/2456949

**Published:** 2018-08-16

**Authors:** Jakrin Kewcharoen, Klaorat Prasongdee, Supanee Sinphurmsukskul, Sarawut Siwamogsatham, Sarinya Puwanant, Pat Ongcharit, Vichai Benjacholamas, Aekarach Ariyachaipanich

**Affiliations:** ^1^Division of Cardiovascular Medicine, Department of Medicine, Faculty of Medicine, Chulalongkorn University, Bangkok, Thailand; ^2^Cardiac Center, King Chulalongkorn Memorial Hospital, Thai Red Cross Society, Bangkok, Thailand; ^3^Excellent Center of Organ Transplantation, King Chulalongkorn Memorial Hospital, Thai Red Cross Society, Bangkok, Thailand

## Abstract

Primary cardiac myxoma is the most common primary cardiac tumor. Tumor resection is the treatment of choice and overall long-term prognosis is good and recurrence is rare. This report presents a case of a young girl who presented with multiple recurrent cardiac myxoma. She underwent 3 sternotomy surgeries of 3 separated episodes of cardiac myxoma resection. On the fourth recurrence, the patient underwent orthotopic heart transplant. The patient tolerated the procedure well and is alive 6 months after the procedure with NYHA class I. We reviewed evidences and summarized reported cases of orthotopic heart transplant operation for primary cardiac tumor in the literature.

## 1. Introduction

Primary cardiac myxoma (CM) is the most common type of primary cardiac tumor with an estimated incidence around 0.5-1.0 per million cases per year [1]. Most of CM is sporadic and located in the left atrium (LA). Patients typically present with obstructive cardiac symptoms and nonspecific systemic signs [2]. Two of the most feared consequences of CM are systemic embolism and postoperative recurrence. Studies found that the recurrence rate of CM is around 2-7% [1, 3, 4]. The incidence of recurrent CM was found more commonly in Carney complex and patients who were presented with multiple lesions, positive family history, younger age, and incomplete resection [3, 5]. The standard of care for CM is surgical excision of the tumor. However, the treatment for patients with recurrent CM is unclear.

Orthotopic heart transplantation (OHT) is a treatment choice primarily reserved for patients with end-stage heart failure due to causes such as viral, ischemia, valvular, or idiopathic [6]. Nevertheless, OHT for primary cardiac tumor had also been reported. Indications for OHT in patients with primary cardiac tumor include unresectable benign or malignant tumor. We present a case of a recurrence CM who was treated with cardiac transplant and a literature review of OHT for primary cardiac tumor.

## 2. Case

**Figure 1:**
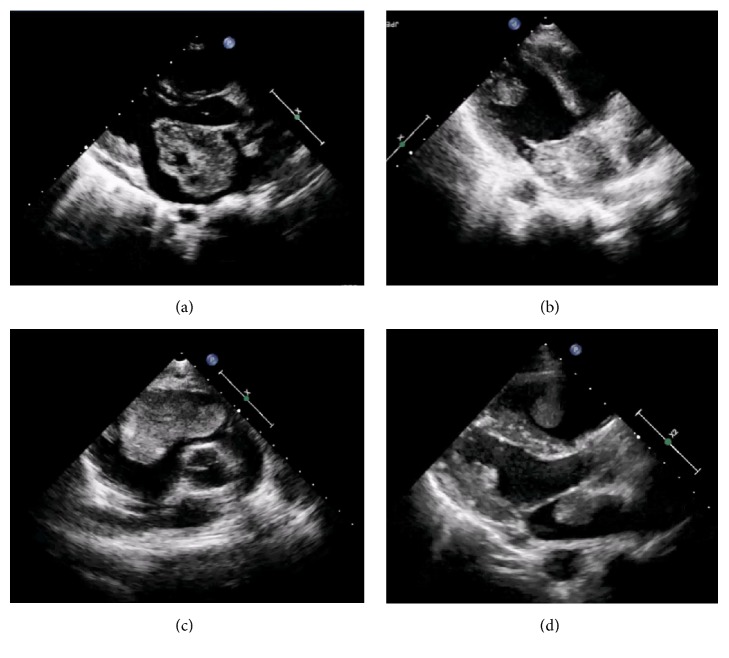
Still images of echocardiogram form the initial diagnosis and subsequent recurrent CM, panel (a) showing a LA mass during initial diagnosis; panel (b) showing LA and LV masses in the first recurrent episode; panel (c) showing an RV mass in the second recurrent episode; panel (d) showing LA, LV, and RV masses in the third recurrent episode.

A 17-year-old girl with a recurrent cardiac tumor presented for the heart transplant evaluation. Initially, at the age of 10, she first presented with dyspnea and holosystolic murmur at apex. She did not have any significant medical history, other than a first-degree relative family history of CM. Echocardiography revealed a 3.5 × 4.8-cm LA mass. The mass was mobile, heterogeneous, and protruded from atrial septum into LA, suspected for CM and causing mitral regurgitation (Figure 1). The patient underwent tumor removal surgery via midline sternotomy. The pathology showed myxoid stroma with clusters of spindle cells and small blood vessels confirming the diagnosis of CM.

A year after the surgery, at the age of 12, she presented to the emergency department with sudden abdominal and right leg pain. The abdominal examination showed left upper abdominal tenderness and guarding. There were also signs of arterial occlusion in her right leg. Echocardiogram showed multiple cardiac masses in LA (2.5 × 1.9 cm) and left ventricular (LV) (1.8 × 1.0 cm) (Figure 1). A computed tomography of the abdomen and lower extremities confirmed the diagnosis of splenic infarction and femoral arterial embolism. She underwent splenectomy and embolectomy. An open heart operation revealed 5 cardiac masses (3 in LA, and 2 in LV) which all were removed. All specimens including the tissue from embolectomy were reported as a CM. The surgery was uneventful and echocardiography afterward did not show tumor residual. The repeat physical examination did not reveal signs of Carney complex.

Unfortunately, at the age of 13, a right ventricular (RV) mass sized 1.7 × 1.6 cm was detected on the echocardiography. She was asymptomatic but with the follow-up echocardiogram showing increasing in size of the mass to 2.8 × 4.7 cm extended into RV outflow tract causing obstruction (peak RV outflow velocity of 3.8 m/s) (Figure 1). After discussion, she underwent a third sternotomy for tumor resection and specimens were reported as CM. The surveillance echocardiography was performed every 6 months and at the age of 15. There were recurrent of cardiac masses in LA, LV, and RV, sized 2.4 cm, 2.1 cm, and 2.4 cm, respectively (Figure 1).

**Figure 2:**
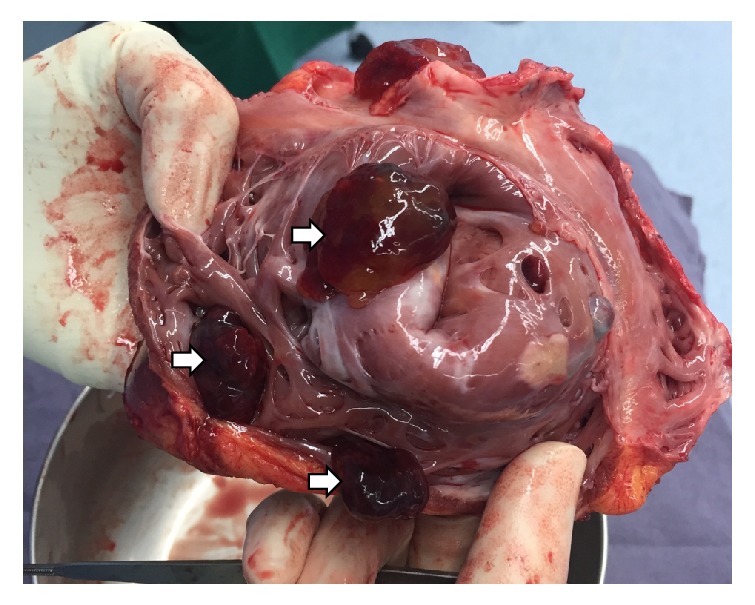
The gross examination of the explant heart showing multiple gelatinous masses (arrows).

Even though CM is considered benign, due to the infrequent recurrence, fast growing, and the complications from the neoplasm including valve regurgitation and obstruction, and embolic event, the heart transplant was proposed with the fourth recurrence of CM. Other than elevated panel reactive antibody with multiple significant anti-human leukocyte antibodies, there were no other contraindications. She was on the waiting list for 11 months. She was treated with warfarin while on the waitlist without new events. Of note, at the time of heart transplant operation, she was 17 years old. The heart transplant was performed with a bicaval anastomosis. Attention was given to maximally excise the recipient cardiac tissue. The gross examination of the excision heart showed 4, 1, and 1 masses in RV, LA, and LV (Figure 2). These masses showed homogenous tan white, gelatinous, rubbery cut surface. The masses were reported as CM.

The clinical course was uneventful. Regarding immunosuppression, she was given standard regimen of our institution for high risk patient including perioperative plasmapheresis and induction therapy with basiliximab, then maintenance regimen with a combination of cyclosporine, mycophenolate, and prednisone. Now, 6 months after the surgery, the patient is doing well with New York Heart Association functional class I. The steroid was weaned off. There was no episode of treated rejection. The physical examination and echocardiogram did not reveal signs of recurrent masses. The echo was planned in a 6-month interval.

The details of each episode of tumor presentation and operations are summarized in Table 2.

## 3. Discussion

CM is the most common primary tumor of the heart. Histologically, it originates from endocardial and consists of myxoid stroma and variable myxoma cells. The most common location of CM is in the LA. Size of the tumor varied from 3 cm to 7 cm [34]. Although the tumor usually presented in a sporadic form, a familial form was also rarely found with most patients presented as Carney syndrome [1]. The tumor itself is histologically benign, but it can cause significant symptoms and morbidity due to a large size and an insecure location. Therefore, patients with CM need aggressive intervention. The gold standard treatment of CM is complete surgical resection of the tumor, with or without the adjacent endocardium tissue. Most of the patients did not require reoperation and prognosis is generally good [2, 3, 35].

Recurrent CM was relatively common in patients with multiple lesions, tumor location other than the LA, family history, and younger age. Possible mechanisms of recurrence were suggested such as incomplete resection, growth from another focus, familial inheritance, or implantation of a fragment from the original tumor. The overall rate of the recurrence was reported to be 2-7% and usually treated with reexcision. In familial type, the recurrent rate was found to be as high as 12% [36]. In our case, based on the young age at presentation and family history of CM, it is likely that the recurrence was due to the inheritance.

However, recurrence for the second time or more is extremely rare and several cases of multiple recurrent of CM had been reported. Herman et al. published a case report of a patient who had three-time recurrence 14 years after the second episode. Conservative treatment of anticoagulant was chosen due to the patient's comorbidity and unwillingness to receive another surgery [37]. Azzam et el. showed a patient with Carney complex who had four recurrent of CM, all in the LA. The patient underwent excision four times and was tumor-free at 36-month follow-up [38].

Although there were cases of recurrent CM that were successfully treated with reexcision, this patient had four recurrent CM and had a high chance of another recurrence. The patient was young and had a family history of CM, which are established risk factors for tumor recurrence. Despite being highly sensitized from previous sternotomy, we believe that OHT may provide better long-term survival. Orthotopic heart transplantation has been considered a final treatment option when others are not available. The use of OHT has been implemented in patients with unresectable cardiac tumor, both benign and malignant. According to the published data, there has been only one case of CM patient who was treated with OHT. Goldstein et al. performed OHT on a patient with recurrent CM due to extensive involvement of the tumor [18].

**Figure 3:**
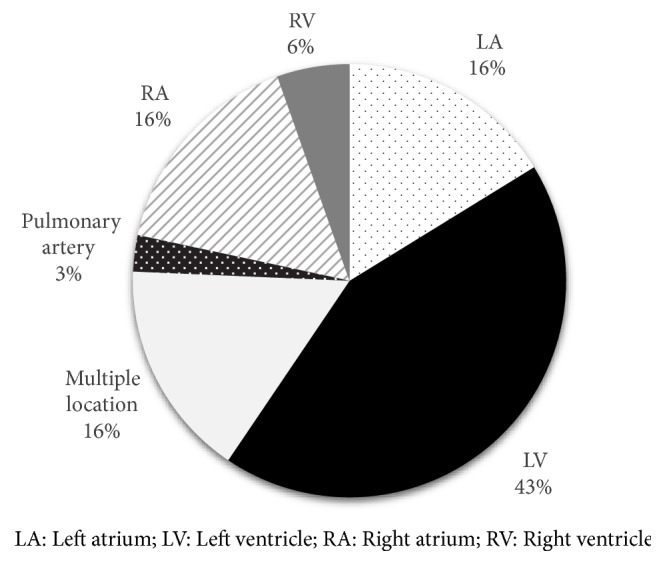
Location of tumors.

**Figure 4:**
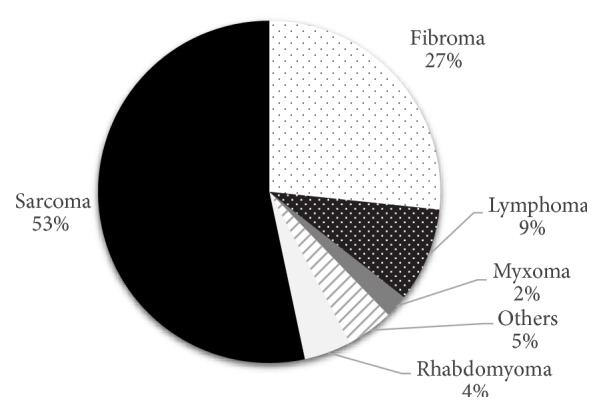
Histology of tumors.

We reviewed patients with a primary cardiac tumor that underwent OHT as a treatment. A review of the literature showed that there have been 46 patients who underwent OHT due to primary cardiac tumor (Table 1). The most common histologic type of tumor was sarcoma (50%) and the most common location of tumors was left ventricle (43%) (Figures 3 and 4). With limited data availability and follow-up time, there was one patient who had a recurrence of the tumor following OHT. Metastasis of the tumor occurred in 11 (23.9%) patients. Mean follow-up time was 29.3 months with a mortality rate of 47.8%.

In summary, heart transplant is one of the treatment options in patient with primary cardiac tumor. This may be considered early, rather than last options, in a tumor type which is highly invasive or has a high likelihood of recurrence.

## Figures and Tables

**Table 1:** Patients undergoing orthotopic heart transplantation for primary cardiac tumor.

Study	Sex	Age (year)	Tumor histology	Location	Metastasis/Recurrence	Follow-up, months	Death
Jamieson *et al.* [7]	F	17	Fibroma	LV	No	75	No

Aravot *et al.* [8]	F	43	Neurofibrosarcoma	RV	No	66	No

Siebermann *et al.* [9]	F	31	Synovial sarcoma	RA, RV	Metastasis	2	Yes

Horn *et al. [10]*	M	13	Angiosarcoma	RA	Metastasis	15	Yes

Mark *et al.* [11]	F	2	Fibroma	LV	No	8	Yes

Aufiero* et al. [12]*	F	31	Fibrosarcoma	LV	No	12	No

Crespo *et al. [13]*	M	31	Angiosarcoma	RA	Metastasis	8	Yes
	M	32	Angiosarcoma	RA	Metastasis	9	Yes

Valente *et al.* [14]	F	1 month	Fibroma	LV	No	36	No
	F	40	Fibroma	LV	No	28	No

Yuh *et al.* [15]	F	57	Lymphoma	LV	Metastasis	14	Yes

Baay *et al. [16]*	M	34	Angiosarcoma	RA	No	33	No

Bachet* et al.* [17]	M	35	Fibrosarcoma	RA, LA	Recurrence	18	Yes

Goldstein *et al. [18]*	F	47	Myxoma	LA	No	18	No

Demkow *et al.*	M	4 months	Rhabdomyoma	LV	No	8	No

Almenar *et al.* [19]	F	29	Angiosarcoma	n/a	Metastasis	2	Yes

Michler *et al. [20]*	F	42	Sarcoma	LA	No	6	No
	F	49	Myxosarcoma	LA	No	34	No
	F	26	Pheochromocytoma	n/a	No	60	No
	F	49	Fibroma	LA	Metastasis	38	No
	F	39	Rhabdomyosarcoma	LV	No	3.5	Yes
	M	3 months	Fibroma	LV	No	105	No

Noirclerc *et al.* [21]	n/a	n/a	Sarcoma	n/a	No	20	No

Gowdamarajan *et al.* [22]	M	64	Sarcoma	n/a	n/a	3	Yes

Grandmougin et al. [23]	M	7	Leiomyosarcoma	n/a	n/a	11.5	Yes
	F	28	Osteosarcoma	n/a	n/a	11.5	Yes
	M	9	Leiomyosarcoma	n/a	n/a	11.5	Yes
	F	61	Histosarcoma	n/a	n/a	36	Yes
	M	8	Lymphoma	n/a	n/a	21	Yes
	M	33	Rhabdomyosarcoma	RA	No	102	No

Talbot et al. [24]	M	40	Sarcoma	RV, PA	Metastasis	48	Yes
	F	39	Sarcoma	PA	No	49	No
	F	37	Sarcoma	LA	Metastasis	16	Yes
	M	45	Sarcoma	LA	Metastasis	5	Yes

Padalino *et al.* [25]	n/a	n/a	Fibroma	LV	No	135	Yes
	F	1 month	Fibroma	LV	No	38	Yes
	n/a	n/a	Fibroma	LV	No	n/a	No
	n/a	n/a	Rhabdomyoma	LV	No	n/a	No

Kobayashi *et al.* [26]	F	7 months	Fibroma	LV	No	36	No
	F	6 months	Fibroma	LV, RV	No	19	No

Agaimy *et al.* [27]	F	36	Sarcoma	RA	No	3	No

Ried *et al.* [28, 29]	F	21	Lymphoma	LV	No	26	No

Fouquet *et al.* [30]	n/a	45	Plasmocytoma	RV	n/a	n/a	Yes

Yang *et al.* [31]	M	53	Lymphoma	RA, RV	Metastasis	12	No

Padalino *et al.* [32]	n/a	n/a	Fibroma	LV	No	42	Yes

Rajakumar *et al.* [33]	M	7	Fibroma	LA, LV	No	18	No

F: female, M: male, RA: right atrium, LA: left atrium, RV: right ventricle, LV: left ventricle, and PA: pulmonary artery.

**Table 2:** Tumor characteristic and operations detail.

No. of surgery (year)	Location and size	Surgical Method	Non-resectable mass	Complication	Residual mass	Pathology
1st surgery (2008)	Echocardiography: Endocardial cardiac mass (3.5x4.8 cm) protruded from LA septum and MV anterior leaflet to LA cavity Intra-op: 5x6x6 cm mass attached to the septum of the LA with no involvement of MV	Open midline-sternotomy for tumor removal	NA	None	Echocardiography (4 days after operation): Tumor remnant (4mm) and mild MV regurgitation.	Myxoma at LA mass. Margin: N/A

2nd surgery (2010)	Echocardiography: LA mass (2.5x1.9cm) obstructing MV inflow. LV mass (1.78x0.97cm) was detected at LV free wall near apex Intra-operation: A 4-cm LA mass attached to LA wall, a 1-cm mass attached to wall of right superior PV, a 0.5-cm mass attached to interatrial septum and 1-cm and 2-cm LV masses	Open midline-sternotomy for tumor removal	None	None	Echocardiography (1 week after the operation): No tumor residual.	Myxoma at LA, right superior PV, lower interatrial septum, LV masses. Clear margin.

3rd surgery (2012)	Echocardiography: RV mass (2.8x4.7cm) from RV apex extended to RV outflow tract. Intra-operation: 5-cm mass at axillary surface and multiple small mass at RV endocardium	Open midline-sternotomy for tumor removal	None	Suspected embolic stroke 1 day after the operation. Patient improved without any intervention	Echocardiography (1 week after the operation): No tumor residual.	Myxoma at RV mass Clear margin.

RA: right atrium, LA: left atrium, RV: right ventricle, LV: left ventricle, PA: pulmonary artery, PV: pulmonary vein, MV: mitral valve, and intra-op: intra-operation.

## References

[1] Lamba G., Frishman W. H. (2012). Cardiac and pericardial tumors. *Cardiology in Review*.

[2] D'Alfonso A., Catania S., Pierri M. D. (2008). Atrial myxoma: A 25-year single-institutional follow-up study. *Journal of Cardiovascular Medicine*.

[3] Wang Z., Chen S., Zhu M. (2016). Risk prediction for emboli and recurrence of primary cardiac myxomas after resection. *Journal of Cardiothoracic Surgery*.

[4] Ansari Aval Z., Ghaderi H., Tatari H. (2015). Surgical Treatment of Primary Intracardiac Myxoma: 20-Year Experience in "shahid Modarres Hospital" - A Tertiary University Hospital - Tehran, Iran. *The Scientific World Journal*.

[5] Selkane C., Amahzoune B., Chavanis N. (2003). Changing Management of Cardiac Myxoma Based on a Series of 40 Cases with Long-Term Follow-Up. *The Annals of Thoracic Surgery*.

[6] Chambers D. C., Yusen R. D., Cherikh W. S. (2017). The Registry of the International Society for Heart and Lung Transplantation: Thirty-fourth Adult Lung And Heart-Lung Transplantation Report—2017; Focus Theme: Allograft ischemic time. *The Journal of Heart and Lung Transplantation*.

[7] Jamieson S. W., Gaudiani V. A., Reitz B. A., Oyer P. E., Stinson E. B., Shumway N. E. (1981). Operative treatment of an unresectable tumor of the left ventricle. *The Journal of Thoracic and Cardiovascular Surgery*.

[8] Aravot D. J., Banner N. R., Madden B. (1989). Primary cardiac tumours - Is there a place for cardiac transplantation?. *European Journal of Cardio-Thoracic Surgery*.

[9] Siebenmann R., Jenni R., Makek M., Oelz O., Turina M. (1990). Primary synovial sarcoma of the heart treated by heart transplantation. *The Journal of Thoracic and Cardiovascular Surgery*.

[10] Horn M., Phebus C., Blatt J. (1990). Cancer chemotherapy after solid organ transplantation. *Cancer*.

[11] Marx M., Buxbaum P., Wimmer M., Laczkovics A. (1991). Heart transplantation in childhood. *Wiener Klinische Wochenschrift*.

[12] Aufiero T. X., Pae W. E., Clemson B. S., Pawlush D. G., Davis D. (1993). Heart transplantation for tumor. *The Annals of Thoracic Surgery*.

[13] Crespo M. G., Pulpon L. A., Pradas G. (1993). Heart transplantation for cardiac angiosarcoma: Should its indication be questioned?. *The Journal of Heart and Lung Transplantation*.

[14] Valente M., Cocco P., Thiene G. (1993). Cardiac fibroma and heart transplantation. *The Journal of Thoracic and Cardiovascular Surgery*.

[15] Yuh DD., Kubo SH., Francis GS., Bank A., McDonald KM., Jessurun J. (1994). Primary cardiac lymphoma treated with orthotopic heart transplantation: a case report. *The Journal of Heart And Lung Transplantation : The Official Publication of the International Society for Heart Transplantation*.

[16] Baay P., Karwande S. V., Kushner J. P., Olsen S., Renlund D. G. (1994). Successful treatment of a cardiac angiosarcoma with combined modality therapy. *The Journal of Heart and Lung Transplantation*.

[17] Bachet J., Banfi C., Martinelli L., Brodaty D., Guilmet D. (1995). Heart transplantation and primary cardiac tumors. *The Annals of Thoracic Surgery*.

[18] Goldstein D. J., Oz M. C., Michler R. E. (1995). Radical excisional therapy and total cardiac transplantation for recurrent atrial myxoma. *The Annals of Thoracic Surgery*.

[19] Rodríguez Blanco V. M., Barriales Álvarez V., Segovia Martínez E., de la Tassa C. M., Barriales Villa R., Llosa A. C. (1996). Reversible dilated cardiomyopathy and thyrotoxicosis. *Revista Española de Cardiología*.

[20] Michler R. E., Goldstein D. J. (1997). Treatment of cardiac tumors by orthotopic cardiac transplantation. *Seminars in Oncology*.

[21] Noirclerc M., Chavanon O., Borrel E. (1997). Primary cardiac sarcoma treated by orthotopic cardiac transplantation. *Archives des Maladies du Coeur et des Vaisseaux*.

[22] Gowdamarajan A., Michler R. E. (2000). Therapy for primary cardiac tumors: Is there a role for heart transplantation?. *Current Opinion in Cardiology*.

[23] Grandmougin D., Fayad G., Decoene C., Pol A., Warembourg H. (2001). Total orthotopic heart transplantation for primary cardiac rhabdomyosarcoma: Factors influencing long-term survival. *The Annals of Thoracic Surgery*.

[24] Talbot S. M., Taub R. N., Keohan M. L., Edwards N., Galantowicz M. E., Schulman L. L. (2002). Combined heart and lung transplantation for unresectable primary cardiac sarcoma. *The Journal of Thoracic and Cardiovascular Surgery*.

[25] Padalino M. A., Vida V. L., Boccuzzo G. (2012). Surgery for primary cardiac tumors in children early and late results in a multicenter european congenital heart surgeons association study. *Circulation*.

[26] Kobayashi D., L'Ecuyer T. J., Aggarwal S. (2012). Orthotopic heart transplant: A therapeutic option for unresectable cardiac fibroma in infants. *Congenital Heart Disease*.

[27] Agaimy A., Rösch J., Weyand M., Strecker T. (2012). Primary and metastatic cardiac sarcomas: A 12-year experience at a German heart center. *International Journal of Clinical and Experimental Pathology*.

[28] Ried M., Hirt S., Schmid C. (2012). Over 2-year disease-free survival after multimodality therapy of a primary cardiac lymphoma. *The Journal of Heart and Lung Transplantation*.

[29] Ried M., Rupprecht L., Hirt S. (2010). Sequential therapy of primary cardiac lymphoma with cardiectomy, total artificial heart support, and cardiac transplantation. *The Journal of Heart and Lung Transplantation*.

[30] Fouquet O., Flecher E., Leurent G., Leguerrier A. (2013). Heart transplantation for cardiac plasmocytoma. *European Journal of Cardio-Thoracic Surgery*.

[31] Yang J., Liu J., Dong N. (2013). Treatment of a primary cardiac lymphoma with orthotopic heart transplantation. *Kardiologia Polska*.

[32] Padalino M. A., Reffo E., Cerutti A. (2008). Medical and surgical management of primary cardiac tumours in infants and children. *Cardiology in the Young*.

[33] Prakash Rajakumar A., Ejaz Ahmed S., Varghese R., Kothandam S., Murmu U. C., Sethuratnam R. (2017). Pediatric heart transplant for unresectable primary cardiac tumor. *Asian Cardiovascular and Thoracic Annals*.

[34] Wang J-G., Y-J Li., Liu H., N-N Li., Zhao J., Xing X-M. (2012). Clinicopathologic analysis of cardiac myxomas: Seven years experience with 61 patients. *Journal of Thoracic Disease*.

[35] Lin Y., Xiao J., Chen J. (2016). Treating cardiac myxomas: A 16-year Chinese single-center study. *Journal of Cardiovascular Medicine*.

[36] Marina K., Vasiliki K. E., George S. (2013). Recurrent cardiac myxoma in a 25 year old male: A DNA study. *World Journal of Surgical Oncology*.

[37] Hermans K., Jaarsma W., Plokker H. W. M., Cramer M. J. M., Morshuis W. J. (2003). Four cardiac myxomas diagnosed three times in one patient. *European Heart Journal - Cardiovascular Imaging*.

[38] Azzam R., Abdelbar A., Yap K. H., Abousteit A. (2014). Carney complex: Fourth time excision of recurrent atrial myxoma via left thoracotomy. *BMJ Case Reports*.

